# Quantum Descriptor-Based Machine-Learning Modeling of Thermal Hazard of Cyclic Sulfamidates

**DOI:** 10.1021/acs.jcim.5c01048

**Published:** 2025-08-15

**Authors:** Michal Dabros, Hagen Münkler, Florence Yerly, Roger Marti, Michaël Parmentier, Anikó Udvarhelyi

**Affiliations:** † Institute of Chemical Technology, Haute école d’ingénierie et d’architecture de Fribourg, HES-SO University of Applied Sciences and Arts Western Switzerland, CH-1700 Fribourg, Switzerland; ‡ Pharmaceutical and Analytical Development, 111826 Novartis Pharma AG, CH-4056 Basel, Switzerland; § Chemical and Analytical Development, 111826 Novartis Pharma AG, CH-4056 Basel, Switzerland

## Abstract

Cyclic sulfamidates are commonly used building blocks in organic synthesis. Correct classification of their thermal criticality is crucial for the safe use of these compounds in process development and scale-up. In this study, building on our earlier work (Ferrari et al., 2022), we focused on modeling the reaction enthalpy of a family of 5-membered cyclic sulfamidates toward strong bases. The key challenge for the modeling task was the sparse availability of measured reaction enthalpies, with only 29 measurements available. To address this challenge, we used descriptors based on the quantum-chemical properties of the molecules, as they are more closely related to reaction enthalpies than typical cheminformatics-based descriptors. This approach allowed us to avoid relying solely on data-to-fit models and to focus instead on modeling reaction enthalpies using chemistry-aware techniques, which are more appropriate for small data sets. Three models were constructed using the quantum-chemical descriptors: the first one combining Partial Least Squares (PLS) regression with a Genetic Algorithm (GA), the second one based on the Least Absolute Shrinkage and Selection Operator (LASSO) method, and last, a Gaussian Process Regression (GPR) model. The three models achieved coefficients of determination of 0.78, 0.67, and 0.74, respectively. Although the absolute prediction error values were close to 100 J/g, it is noteworthy that all three techniques provided similar results and accurately classified nearly all compounds into their respective thermal criticality classes. This highlights the methodology’s effectiveness in providing a reliable framework for preliminary safety assessment and decision-making in process development.

## Graphical abstract

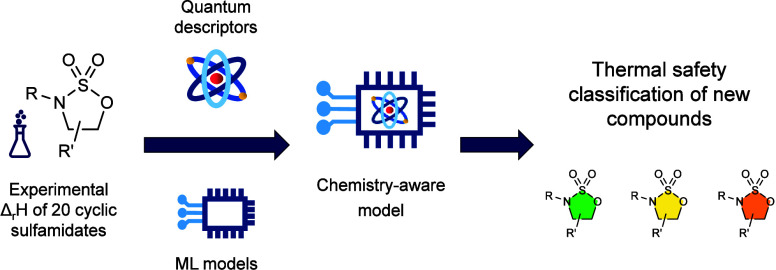

## 1 Introduction

The synthesis of Active Pharmaceutical Ingredients (APIs) or other relevant organic molecules often requires the use of building blocks to install specific functional groups. The more the complexity of the targeted molecule increases, the more the synthetic strategy needs to be elaborated to minimize the numbers of chemical steps while keeping the risks of handling hazardous substances and reactions at a minimum. Cyclic sulfamidates are typically used as alkylating reagents that allow for the synthesis of substituted amino alcohols, diamines, lactams or fluorinated derivatives to name a few, showcasing their high versatility.

The electrophilic character of the sulfamidate ring undergoes regiospecific nucleophilic substitution at the O-bearing carbon center C­(O), releasing sulfur trioxide (SO_3_) together with energy.^2–5^ Interestingly, the reactivity of five-membered sulfamidates is strongly modulated by the substitution/protection at its ring-N atom and its C terminus.^2,3^ The substituent effect has been investigated in detail by Navo et al. through combined experimental and transition-state quantum-chemical calculations including reaction activation energy barrier calculations, to support peptide synthesis.^2^

Along with the modulation of the sulfamidate reactivity (switching reactivity at the quaternary center or fine-tuning the chemoselectivity^2^) come as well largely different associated thermal risks. We recently reported the thermal risk assessment of several 5-membered cyclic sulfamidates supported by property relationship modeling and chemometric modeling of their thermal properties.^1^ Specifically, we investigated the reaction enthalpy toward strong bases, and we observed that the thermal behavior was highly dependent on the individual compound’s substitution groups. The goal of the study was to develop a simple modeling approach to predict the thermal behavior of the individual sulfamidate as a function of the molecular structure alone, without exact reaction understanding, in order to provide early risk assessments and go/no-go decisions during screening or process development. The desired risk assessment should not involve an elaborate reaction path quantum-chemical assessment as in the case of Navo et al.;^2^ first because we might not know the exact reaction in all cases, and second because we need to provide a fast and simple assessment in an industry scenario that we cannot realize with transition-state calculations. Building on our previous work,^1^ here we present simple models to realize this structure-based risk assessment.

The possibility of correlating structural features to reactivity is already exemplified by numerous data-driven strategies that are based on linear free energy relationships (LFERs), an approach first introduced by Hammett on reactions with benzene derivatives.^4^ LFERs may provide mechanistic insights and predict reactivity in unknown reactions in simple cases. The limited prediction power in complex systems is systematically addressed by the development of more complex multiparameter approaches during the past decades to better correlate chemical reactivity with structure. A comprehensive review of how data-driven modeling in chemistry evolved over the past decades is presented by Williams et al.^5^

The success of any data-driven model depends on suitable molecular and reaction representations that translate the chemistry to a format that can be handled by the computer. For a complete, accurate model of a molecular system one would need to solve the Schrödinger equation for the molecular electronic Hamiltonian representing the total electron density of the system. The reactions would need to be modeled by full transition-state calculations using the molecular electronic Hamiltonians of all reactants. Computationally such calculations are prohibitively expensive for any practical molecule, hence numerous strategies have been developed to make the problem accessible for lower-cost approaches.

From the molecular electronic Hamiltonian, certain properties may be derived that can be used to suitably represent a molecule with respect to a certain behavior of interest in a model. Such derived properties are referred to as quantum descriptors or features and may be provided as input for various machine learning (ML) models. Most abundantly used are quantum descriptors derived from density functional theory (DFT) calculations, being the most popular quantum-chemistry theory. We refer to this type of models as quantum-descriptor based ML models or ML models using quantum-chemical features.

As the Schrödinger equation is general, quantum descriptors offer transferable representations of the compounds by construction and hence one could expect good extrapolation capabilities of models using them. However, human insight and expert knowledge are required to select upfront the relevant quantum descriptors for the problem statement. There is currently no standard which descriptors (and which particular implementation of a descriptor) to use, hence they are often also referred to as expert-guided features. Another disadvantage of using them as molecular representations might be that quantum-chemical calculations require 3D conformers and geometry optimizations. Although today’s DFT software implementations are highly efficient and geometry optimizations are by far not as expensive as full reaction-path calculations, the geometry optimizations still are not “instantly” available.

An alternative approach to represent molecules for ML models is to use fingerprints or graphs, which do not require any explicit physical information or expert knowledge and are fast to generate. These representations have been successfully applied in various chemistry problems.^6^ However, models using these representations cannot extrapolate well to novel structures and they are highly dependent on the quality, amount and diversity of the data, as well as biased to limited molecular and property ranges. To achieve practically useful accuracies, one needs vast amounts of data to fit models using such molecule representations. The reactivity prediction studies by Götz et al, used 40,000 reactant combinations in 50,000 reactions.^7^ Interestingly, the authors recommend using dedicated “local” models with a given sparse data set for reliable predictions as opposed to building a general model for chemical reactivity.^7^ In summary, feature engineering and development of molecular representations will certainly be continuing. For an extensive and comprehensive review of molecular representations the reader is referred to Sanchez-Lengeling and Aspuru-Guzik.^8^

Recently, several studies have been published that have successfully developed quantum descriptor-based ML models. For example, Christensen et al.^9^ and Haas et al.^10^ have successfully developed ML models for Suzuki-Miyaura optimization and amide coupling reactions, respectively, that rely on quantum descriptor sets as molecular representations, such as atomic charges, frontier orbital energies, and Fukui functions. They demonstrated that their models could accurately predict the reaction yields and selectivities for a large and diverse set of substrates and catalysts with the quantum descriptors being required for the models. Another example is the work by Guan et al.,^11^ who used on-the-fly calculated quantum descriptors to predict the site-selectivity in electrophilic aromatic substitution reactions. A recent study by Shimakawa et al.^12^ also showed interesting efforts in using quantum descriptors to improve the extrapolation ability of ML models to predict molecular properties, such as boiling and melting points or acidic and basic dissociation constants, even in small-data cases with only 200–500 available data points. Another noteworthy example is the study by Finkelmann et al.,^13^ where the authors demonstrated the usefulness of quantum-chemical descriptors for ML models in site of metabolism prediction. In an industry setting, Ertl et al. review quantum-descriptor based ML models in drug design problems.^14^ A most recent example by Gandhi et al. presents a successful reaction condition prediction study involving primary sulfamidates using a set of quantum-chemical descriptors in different ML models, similar to our current approach.^15^

Parallel to these advancements, some studies dampen the enthusiasm of quantum descriptor-based ML models. Šícho et al.^16^ for example show that for modeling the regioselectivity of cytochrome P450 enzymes a simple, circular descriptions of atoms and their neighborhood with 2D descriptors can yield models that are robust and just as accurate as models with quantum-chemical descriptors. A recent study presented by Keto et al.^17^ comes to similar conclusions with their study on Diels–Alder reaction outcome prediction, using a data set size of above 6000 data points. They showed that their generative graph-based models (a model that has connectivity information and hence can be regarded a chemistry-aware model) are not improved when incorporating quantum-descriptors. They explain the finding by quantum features being potentially too noisy or the model already having latent understanding of those features. In summary we conclude that the use of quantum-chemical features in ML models needs to be closely aligned with the problem statement and expectations; in certain cases they are crucial,^13,15^ in others they might even be redundant.^17^

In addition to reactivity problems, the application of ML techniques in modeling thermal properties of chemical compounds has attracted significant attention in recent years, exploring a powerful tool for safety assessment and hazard classification studies. The literature presents a wide range of studies reporting the diverse methodologies and applications of ML in predicting thermal properties. Strieth-Kalthoff et al.^18^ provide a comprehensive review, offering insights into the integration of ML techniques across synthesis planning, property estimation, molecular design, and reactivity prediction. This sets the stage for understanding the broader context of ML’s impact on synthetic chemistry. Other reviews^13,14^ discuss the concept of quantitative structure–property relationships (QSPR), presenting various methodologies for developing predictive models for estimating melting points, boiling points, critical temperatures, heats of vaporization and formation, among others. Various studies involving QSPR methodologies are reported for predicting thermal stability, explosivity limits, heats of decomposition, and flammability,^15–17^ and for classifying compounds in terms of thermal risk.^18^ Duan et al.^19^ delve into the prediction of thermal decomposition temperatures of ionic liquids, employing norm indexes and GA-enhanced multiple linear regression (MLR) modeling. A reaction enthalpy prediction study using quantum-chemical calculations was presented by Sayyed et al. for a range of compounds, in certain examples successfully correlating simple quantum descriptors with the reaction enthalpy itself.^20^

In this study, a major limitation for the modeling approach was the small data set size of only 29 compounds (shown in Figure 1), which was due to synthesis challenges and scarce commercial availability. (The full list of compounds considered in the study is presented in the SI.) This represents a completely different scale compared to other “small-data” studies which typically involve hundreds.^12^ To overcome this challenge, we based our molecule representations on relevant quantum-chemical properties of the compounds, such as selected partial charges, Fukui indices or the energies required to break certain bonds relevant to the reaction at hand. The idea of relying on these properties to represent the compounds in a model is that the mapping between them and the reaction enthalpy could be simple enough to extract even from a small data set. For a cheminformatics-based representation that is based on the presence or absence of certain functional groups, this would not be expected. In other words, we complement the small data size with physics-based descriptors relevant to the reaction.

**Figure 1:**
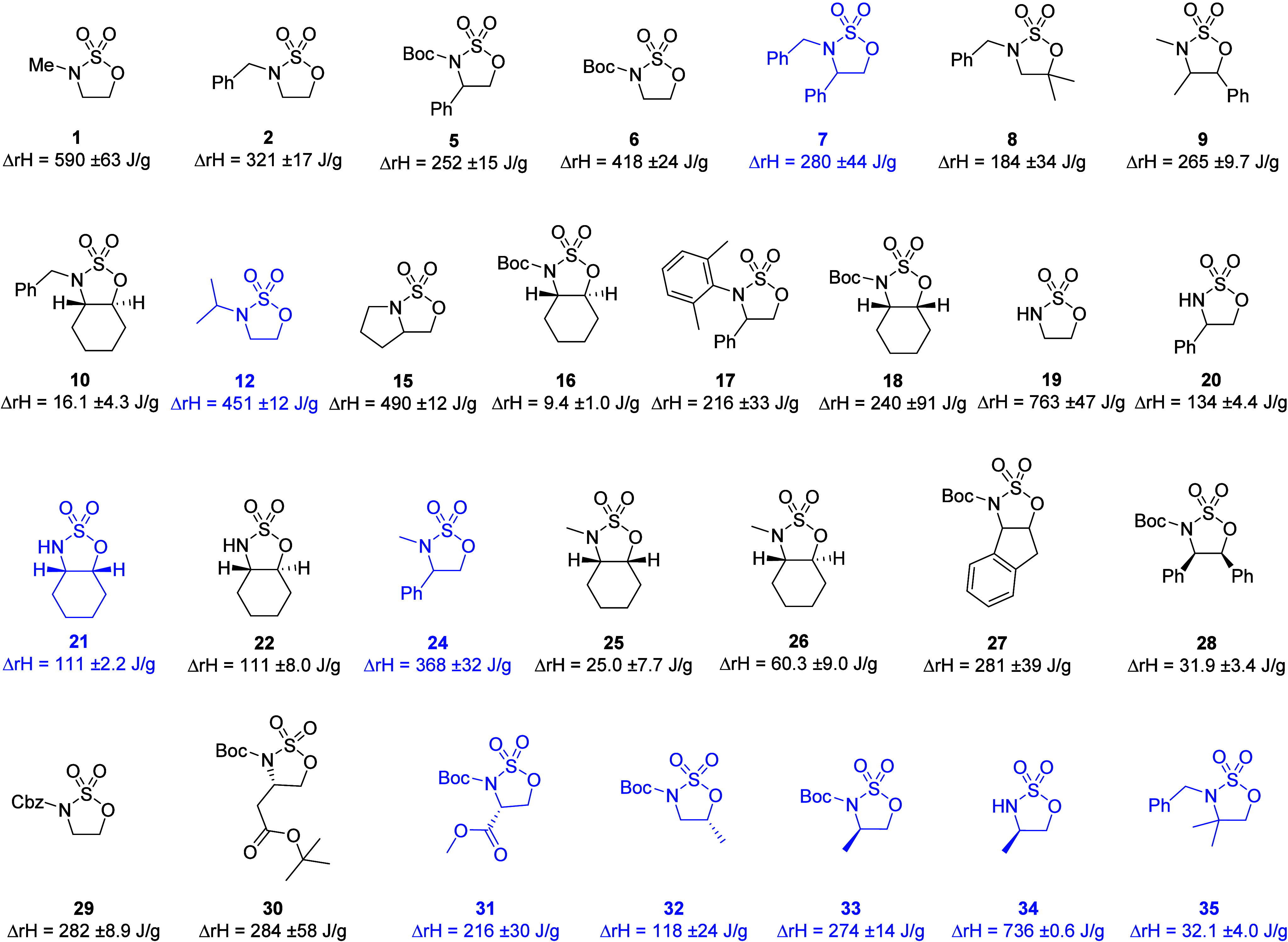
Library of 5-membered cyclic sulfamidates investigated in this work. Compounds marked in black were used as the training set for building the models, while those in blue were used as the prediction set to test the models.

Using an in-house Python package to orchestrate all the needed quantum-chemistry calculations, we obtain a representation of the compounds in terms of 71 quantum-chemical properties. Subsequently, a Partial Least Squares (PLS) model, optimized with a Genetic Algorithm (GA), a Least Absolute Shrinkage and Selection Operator (LASSO) model and a Gaussian Process Regression (GPR) model were built, linking a collection of these QC-based molecular descriptors to the reaction enthalpy values measured with a tailor-made mini-calorimeter. The selection of descriptors was guided by chemical knowledge of the reaction, aligning with previous studies. The application of this knowledge in the models are discussed in the following sections.

## 2 Methodology

### 2.1 Cyclic Sulfamidates and Reactivity Measurements

A library of 29 N- and ring-substituted five-membered cyclic sulfamidates were studied in this work.^1^ The enthalpy of reaction between each compound and the strong organic base 1,8-diazabicyclo[5.4.0]­undec-7-ene (diazabicycloundecene, DBU) in acetonitrile, was measured using a custom-made, milliliter-scale reaction calorimeter.^21^ The vessel had a working volume of 0.5–2.0 mL and was equipped with a Type K thermocouple and a magnetic stirrer. The reactant was added instantaneously by syringe, and the temperature profile was recorded at a 0.5 s interval until reaction completion. The reaction enthalpy was calculated using a previously established and validated heat balance. Each measurement was performed three times to confirm repeatability and obtain a mean value. Enthalpy values ranging from 9.4 ± 1.0 to 763 ± 47 J/g have been measured. Figure 1 shows the structures and the measured enthalpy values for each of the molecules.

As shown in Figure 2, the main reactivity of cyclic sulfamidates with nucleophiles and bases is ring opening by attack on C­(O) via a classical S_N_2 pathway, followed by SO_3_ release upon hydrolysis.^22–24^ Depending on the ring substitution pattern as well as the strength of the base, other mechanisms could be observed, such as elimination reaction E2 and extrusion of SO_3_ to form double bonds in the case of tertiary substituted C­(O) derivatives,^2,25^ or the deprotection of carbamate groups on N.^26^ The presence of electron-donating or withdrawing functional group is expected to impact the reactivity of the cyclic sulfamidate toward a strong base such DBU. Incorporating the electronic character of the carbon center next to the nitrogen and oxygen atoms is therefore crucial to build a good model. Based on this reaction understanding and the knowledge gathered during our previous study,^1^ we selected appropriate atom, bond and molecule-specific descriptors to build our thermal safety prediction models.

**Figure 2:**
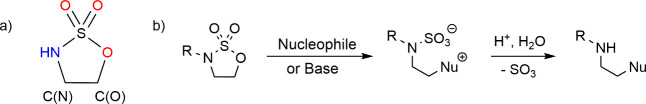
(a) General structure of the 5-membered cyclic sulfamidates studied. (b) 5-membered cyclic sulfamidate supposed degradation reaction pathways considered for the study.

### 2.2 Compound Descriptors

For the small data set at hand, it is imperative to find an appropriate set of descriptors that describe the reactivity of the compounds accurately in terms of relatively few descriptors. In our first study we already showed that quantum-chemical descriptors may capture the reactivity behavior of the molecule and allow to correlate structure with thermal properties to some degree.^1^ Here, we present a more systematic and thorough investigation of the different descriptors and show how the models can be further improved with them. We present an overview of the property calculations below; a detailed description can be found in the Supporting Information (SI).

First, we note that the QC properties of a compound depend on its conformation. Depending on the property in question, this dependence can be more or less significant.^27,28^ We thus rely on averaging and determine the properties by Boltzmann averaging over relevant and representative conformer ensembles. To obtain this representative conformer ensemble for all compounds and their degradants, we rely on the ReSCoSS workflow previously developed by us.^29^ All molecular descriptors are calculated for each of these conformers in acetonitrile solution in line with the experiment, and finally Boltzmann weighted. To calculate the properties themselves, we have applied both semiempirical methods (GFN2-xtb)^30^ and DFT-based approaches using both Turbomole^31^ and Schrödinger Jaguar.^32^ The general structure of the cyclic sulfamidate with the common five-membered ring of the compounds is shown in Figure 2a. Since we know that the reaction proceeds with a ring-opening of this sulfamidate moiety, the most important descriptors are atom- and bond-level properties of this ring.

As a bond-specific property, we consider the dissociation energies for the hydrogen atoms attached to the carbon atoms C­(O) and C­(N) in the common ring. These can be calculated using a workflow available in Schrödinger Jaguar.^32^ If the reaction involves the abstraction of a hydrogen atom at an initial step, it is plausible that the energy required would be linked to the reaction enthalpy in some way. We note that the bond dissociation energy cannot be defined for C­(O) in Compound **8** and for C­(N) in Compound **35**, since there are no hydrogen atoms connected to the respective carbon atoms in these compounds. More details on how these missing values were handled in the modeling step are presented below. Additionally, we consider the bond order between the oxygen atom in the ring and the adjacent carbon atom, given that this bond is broken in the assumed reaction, as shown in Figure 2b.

As atom-specific properties, we consider partial charges and Fukui indices.^33,34^ These are commonly used QC descriptors for reactivity prediction.^11,35^ The Fukui functions consider the change in electron density between neutral and charged states. Different versions are considered for electrophilic, nucleophilic and radical attack, which each compare different charged states. For the Fukui indices, these densities are condensed to the nuclei of the compound.

For each of these properties we obtain eight descriptors, one for each heavy atom within the ring in Figure 2a as well as for the hydrogen atoms bonded to these atoms. In case there are several hydrogen atoms bonded to one of the heavy atoms, we consider the average value of the property. If there is no hydrogen atom present, the respective missing values are handled in the same way as for the bond dissociation energies discussed above. In addition to the reactivity of a given center, steric hindrance is often considered when modeling reactivity. To account for that, we have included the Labute accessible surface area, a classical cheminformatics-based descriptor, and a custom-developed descriptor termed “visible sky”: Given the three-dimensional structure, we calculate which part of the full solid angle is obstructed by other atoms when looking from the center of the atom in question.

In addition, we employ a collection of QC properties, such as the HOMO–LUMO gap or the dipole moment for the whole molecule and for each of its degradants. Lastly, we consider the difference in free energy between the compounds and the degradants arising from the reaction with the base DBU in acetonitrile (Figure 2b). This updated, more thorough approach selecting QC properties resulted in 71 descriptors in total for each compound, all of them conformer-averaged, as opposed to the original 38 descriptors in our last work^1^ using only single conformations.

The calculation of all descriptors described above is automated in a Novartis-internal Python package, which facilitates using them as input to a machine learning model. Since the package is versioned, we can rely on a fixed version to ensure that the descriptors are calculated in the same way as they were for the training data.

In light of recent work on using ML models to predict properties at DFT-level, it would also be interesting to explore an equivalent approach, which switches our QM descriptors for the respective ML-based ones, thus allowing near-immediate inference. In our case, most of the necessary descriptors could be extracted from the “alfabet”^36^ and “qmdesc”^11^ packages, which were both trained using large amounts of DFT calculations. Such approaches become even more important when the DFT calculations are not affordable or fast inference is needed.

### 2.3 Machine Learning-Based Modeling of Thermal Properties

Three types of models have been developed, trained, and evaluated in their ability to predict the thermal behavior of the sulfamidates as a function of the compound descriptors. First, a Partial Least Squares (PLS) model was created and optimized using a Genetic Algorithm (GA) approach that sought to minimize the cross-validation error by selecting the optimal set of descriptors. In the second model, we apply a Least Absolute Shrinkage and Selection Operator (LASSO) regression model for its ability to select significant descriptors automatically. The third model is based on Gaussian Process Regression (GPR), a nonparametric, probabilistic machine learning technique used for regression tasks. The three statistical learning approaches are described in more detail in the following subsections.

The library of sulfamidates was split into a training set, containing 20 compounds, and a prediction set, containing 9 sulfamidates (compounds **7**, **12**, **21**, **24**, **31**, **32**, **33**, **34** and **35**, shown in blue in Figure 1). The training and prediction sets were selected in such a way as to include within each of the two sets a representative sample of the compounds, in terms of both the molecular structure and the reaction enthalpy values. For parameter tuning of the models, a leave-one-out cross-validation on the training set was performed. The prediction set was only used to evaluate the final models.

The two missing values in the data set were substituted using the average values of the two descriptors in all the compounds comprising the training set. Thus, 102.7 was used for the BDE C­(O)–H descriptor in Compound **8**, and 99.1 was used for the missing BDE C­(N)–H descriptor in Compound **35**. Initially, using an artificially high value of the descriptors was tested, following the logic that since the corresponding C–H bonds did not exist in the two compounds, the bond dissociation energy should be, in principle, infinite. However, it was found that introducing abnormally high values in the data set effectively weakened the performance of the model. To explain this effect, note that in a linear model an artificially high value of a feature leads to that feature having a large influence on the prediction value, whereas we are looking for the opposite effect: obtaining a prediction without taking this feature into account for that compound. In the absence of a model that can handle missing values, using the mean value comes closest to this objective. Therefore, though less intuitive from the chemist’s perspective, average values were used for the missing descriptors.

The performance of the models was assessed from two angles: (i) the numerical precision of the enthalpy prediction, quantified by calculating the standard error of prediction; and (ii) the correct classification of the compounds in the four thermal criticality classes defined by Stoessel.^37^ The numerical precision of each model was evaluated using leave-one-out cross validation on the training set. Once built and trained, the models were applied to estimate the enthalpies of the nine compounds in the prediction set. The standard errors of cross-validation (SEV) and the standard error of prediction (SEP) were calculated using the root-mean squared error formula:

$$\mathrm{RMSE}=\sqrt{\frac{1}{n}\sum_{i=1}^{n}({\hat{y}}_{i}-y_{i}{)}^{2}}$$

where *y*_*i*_ is the measured enthalpy of compound *i*, ${\hat{y}}_{i}$ is the enthalpy predicted by the model, and *n* is the number of compounds in the cross-validation or the prediction sets. For the evaluation of the precision of the models, the enthalpies are considered in J/g, given that the thermal criticality classes are defined accordingly. For the classification exercise, we used Stoessel’s definitions: the green, yellow and orange colors in the Figures below represent the negligible (<100 J/g), medium (100–400 J/g) and critical (400–800 J/g) thermal classes, respectively;^37^ none of the compounds that were studied fell within the catastrophic class (>800 J/g).

#### 2.3.1 Partial Least Squares and Genetic Algorithm

Partial Least Squares (PLS) is a chemometric modeling technique that finds a linear regression matrix between a set of independent variables (in this case, the descriptors) and a set of dependent variables (here, the measured enthalpies) with the assistance of Principal Component Analysis (PCA). The two data sets are first decomposed, simultaneously, into a series of latent variables called principal components (PCs) that best summarize the variation in the data in terms of least-squares. A regressor matrix is then sought to maximize the covariance between the latent variables describing the dependent variables and those describing the independent ones.^38–40^ PLS regression surpasses simple multiple linear regression (MLR) in its effectiveness when handling noisy data with numerous predictor variables characterizing complex systems with limited measurements.^38,41^

The optimal number of principal components used by a PLS model is usually selected by assessing the percentage of variance captured by each PC in the modeled data, with caution taken to avoid overfitting the model. In this study, the number of principal components was chosen to capture 85% of the total variance in the descriptors data set. All data were mean-centered and variance-scaled prior to applying PCA and calculating the regression matrix. The original (unoptimized) PLS model was built and tested using the full set of 71 descriptors. To enhance the model’s performance, a Genetic Algorithm was subsequently applied to select the optimal set of descriptors that resulted in the lowest standard error of leave-one-out cross validation.

Genetic Algorithms are an evolutionary optimization method that apply mechanisms akin to those found in genetics and natural evolution to solve process optimization problems.^42–44^ This technique is well-suited for variable selection optimization in multivariate modeling and is frequently applied for this purpose in the area of spectroscopy.^45–48^ Duan et al.^19^ applied a Genetic Algorithm to help in norm index selection for a MLR model predicting the thermal decomposition temperatures of ionic liquids. Indeed, the algorithm’s use of binary solutions (represented by zeros and ones) renders it ideally suited for variable selection in modeling applications.

In this study, the descriptor-selection problem is defined as follows. A population of candidate solutions (in this case, candidate sets of descriptors to be used to build the PLS model) are coded into chromosomes (sequences of zeros and ones) and manipulated by three major operations: mutation, crossover and purge. The crossover process involves selecting two parent chromosomes and randomly swapping bits between them, generating a new chromosome or offspring. The mutation operation is applied by changing randomly selected bits, thereby introducing genetic alterations to the population. Finally, during the purge step, the algorithm discards the weakest chromosomes in the population and substitutes them with new, randomly generated ones. The objective function (here, the standard error of cross-validation) is calculated for each candidate solution by building and evaluating the PLS model that uses the descriptors encoded in the corresponding chromosome. The best solution (ideal set of descriptors) emerges over the course of multiple generations in a manner that is analogous to the survival of the fittest in nature. The size of the population and the number of generations (algorithm iterations) needs to be chosen appropriately to maximize the method’s chance of reaching the optimal solution.^44,49^ In this work, a population of 3550 chromosomes was used (corresponding to 50 times the number of variables), and 1420 generations of the algorithm were run (20 times the number of variables) to achieve convergence at the lowest value of SEV. The crossover rate was kept at 70%, the mutation rate at 4% and the purge rate at 20%. The PLS model, as well as the Genetic Algorithm routine, were programmed in the Matlab environment (The MathWorks Inc., Natick, MA, USA). Following the optimization step, the final model was applied to estimate the enthalpies of the 9 compounds in the prediction set, as shown in Figure 3.

**Figure 3:**
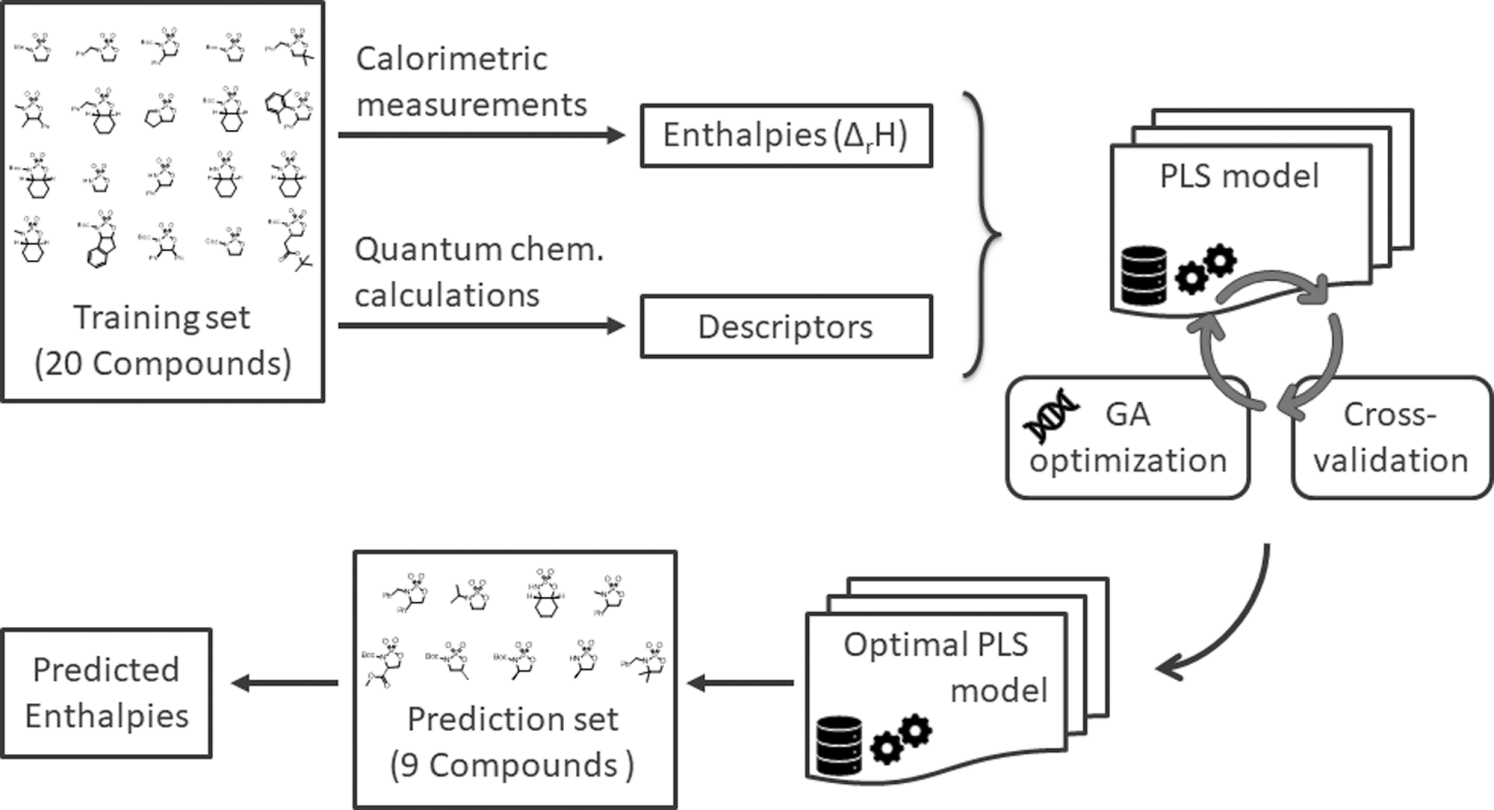
Overall approach applied in the development and optimization of the GA-enhanced PLS model.

A similar methodology has already been investigated in previous work,^1^ yielding encouraging results. In this study, we have increased the number and the complexity of the descriptors. In addition, the division between the training and prediction sets has been adjusted by expanding the prediction set from four to nine compounds. This adjustment ensures that the compounds selected for the prediction set offer a representative sample of all the molecules within the library, encompassing both molecular structure diversity and a full range of reaction enthalpy values.

#### 2.3.2 LASSO Model

The Least Absolute Shrinkage and Selection Operator (LASSO) model is a modification of the classical ordinary least-squares regression.^50^ In traditional linear regression, the goal is to minimize the sum of squared differences between the predicted values and the actual values. However, in the presence of a large number of features (here the 71 descriptors), the model is no longer suitable and does not correctly predict the new data.

The LASSO regression addresses this issue by adding a penalty term to the linear regression objective function. The penalty is based on the absolute values of the regression coefficients. This penalty encourages some coefficients to be exactly zero, effectively performing feature selection by excluding certain descriptors from the model. The method is effective for automatically selecting the most important descriptors.^51^

The LASSO regularization term is controlled by a hyperparameter, often denoted as lambda (λ). As the value of λ increases, the regularization effect strengthens, leading to more coefficients being shrunk toward zero. By tuning the λ parameter, one can control the balance between model simplicity (fewer features) and accuracy. While LASSO can help reduce overfitting by discouraging overly complex models, improper tuning of λ may still lead to underfitting or overfitting. The hyperparameter λ was selected using leave-one-out cross-validation, by identifying the value that resulted in the lowest mean squared error (MSE). All the LASSO computations were done in R (The R Foundation for Statistical Computing, Vienna, Austria) and the package glmnet.^52^ Data were mean-centered and variance-scaled (based on the respective training set) prior to the modeling step.

#### 2.3.3 Gaussian Process Regression

Both of the modeling approaches presented above rely on optimizing the selection of descriptors in the cross validation run on the training set. Thus, they potentially allow to overoptimize during the cross-validation, further tuning parameters while the real performance of the model, as measured on the hold-out test set, no longer improves. This effect is distinct from overfitting since the test compounds in the cross-validation are not seen during the training of the model for the given fold. Given the small size of our data set, our options to avoid this effect are limited. The approach presented below relies on doing few evaluations of the SEV. To achieve this, many aspects of the modeling work were motivated by the experience of the authors and not all choices could be explored within the cross-validation.

As a model we considered Gaussian Process Regression (GPR), a nonparametric, probabilistic model, relying on a choice of kernel function to describe the proximity of different points in the feature space. For an introduction to the use of Gaussian Processes as a regression model, the reader is referred to a paper by Becker.^53^ The computations were done in Python, using the popular machine-learning package scikit-learn.^54^

The model differs from the two models presented above in the way the target reaction enthalpies are presented to the model: they are provided per mol of the considered compound rather than per gram as for the other models. The motivation for this was the assumption that the reaction enthalpy per molecule could be related to the QC-based descriptors in a simpler way. Essentially, the model would not have to learn the weight of the molecule to predict the reaction enthalpy per gram.

In the cross-validation run on the training set, both the kernel function and the selected features were varied. For both aspects, only a limited set of options was explored. The feature selection was done as follows: Picking from the descriptors with the highest Pearson correlation with the reaction enthalpy on the training set, diverse sets of features were selected, making sure both reactivity-related descriptors and such describing steric hindrance were included and not considering descriptors which were deemed too similar to each other (for details, the reader is referred to the SI). Thus, only a few different combinations of molecule descriptors were tried, essentially only varying the number of descriptors chosen. The final model, with the best performance in the cross-validation run, relies only on six descriptors: two Fukui indices, two partial charges and two further descriptors related to steric hindrance, cf. Table 1.

**Table 1:** Descriptors Selected by the Three Modeling Methods

descriptor	PLS-GA	LASSO	GPR
xtb fukui plus N	X	X	
xtb fukui plus C(N)H	X		
xtb fukui plus C(O)	X		X
xtb fukui plus C(O)H	X	X	
xtb fukui zero NH	X	X	
xtb fukui zero C(O)		X	X
xtb fukui minus C(O)	X	X	
xtb mulliken charge O			X
xtb mulliken charge C(N)H	X	X	
xtb mulliken charge C(O)			X
xtb mulliken charge C(O)H		X	
jaguar fukui NN HOMO N	X		
jaguar fukui NN HOMO C(N)H		X	
jaguar fukui NN LUMO N	X	X	
jaguar fukui NN LUMO NH	X	X	
jaguar fukui NN LUMO C(N)H	X	X	
jaguar fukui NN LUMO C(O)H	X	X	
dipole moment (xtb)	X	X	
C(N) H abstraction energy	X		
C(O) H abstraction energy	X	X	
visible sky O		X	
visible sky C(N)			X
visible sky C(O)			X
homolumo gap (xtb) deg 2		X	
DSC Sum dH (kcal/mol)	X	X	

For the kernel function, it was found that the inclusion of a dot-product kernel, which describes the proximity of two vectors in the feature space in terms of the angle between them, produced the best results. Note that also here, the data were mean-centered and variance-scaled (based on the respective training set) before being passed to the model.

## 3 Results and Discussion

### 3.1 Prediction with DSC Data Alone

As a suitable baseline to which we compare the results of our statistical learning models using the QC descriptors, we attempted to predict the reaction enthalpies using solely data obtained with a Differential Scanning Calorimeter (DSC). DSC measurements are routinely conducted in safety laboratories, making them readily available for an initial assessment of a compound’s reactivity potential. Moreover, Sayyed et al. has reported a strong correlation between DSC data and the reaction enthalpy in some cases.^20^

We assumed that the total enthalpy measured across all events detected by the DSC calorimeter within the temperature range of 20–400 °C could be correlated with the observed reaction enthalpy of each compound. Thus, we performed a linear regression on the 20 compounds of the training set, that resulted in a model exhibiting a rather poor performance, with a coefficient of determination *R* ^2^ of 0.127 and a standard error of cross-validation of 181.9 J/g. Applying the model to the nine compounds of the prediction set, a standard prediction error of 176.0 J/g was obtained and three out of the nine compounds (**12**, **31** and **35**) were misclassified, as shown in Figure 4. It is rather clear that such a model cannot reliably be used in the laboratory to guide experiments.

**Figure 4:**
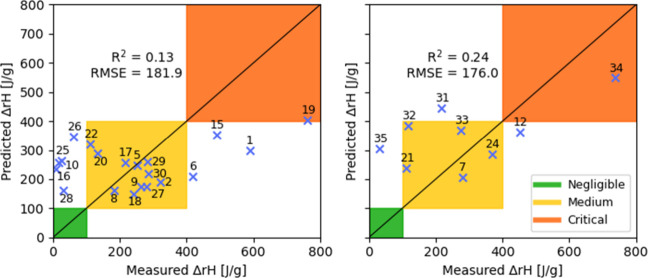
Cross-validation (left) and prediction (right) results obtained using solely DSC data.

Our three rather different statistical learning models, however, yielded much better and comparably consistent results. The reaction enthalpy of the compounds was predicted with a root-mean-square error of approximately 100 J/g in all three cases, thus providing a reasonably accurate preliminary safety assessment for the studied sulfamidates.

### 3.2 PLS-GA Model Results

We first tested the original, unoptimized PLS model using the full set of 71 descriptors. The standard error of cross-validation was 105.0 J/g, as shown in Figure 5 (left), with the enthalpy of several compounds significantly misestimated, and in two cases predicted to be of negative values (those were set 0). Applying this model to the prediction set, a value of 113.3 J/g was obtained for the Standard Error of Prediction (Figure 5, right). All but one of the nine compounds tested in the prediction set, were classified in the correct thermal criticality class – Compound **35** was the only one whose reaction enthalpy class was overestimated by the model.

**Figure 5:**
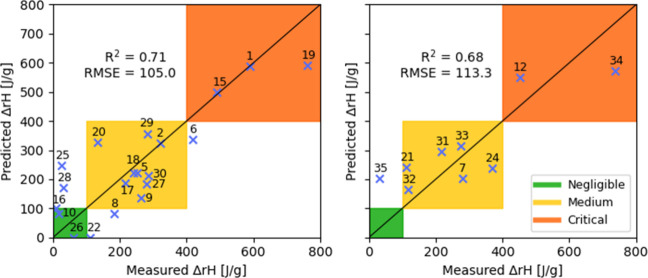
Cross-validation (left) and prediction (right) results obtained using the original PLS model, using the full set of 71 descriptors.

The Genetic Algorithm selected 16 descriptors for the optimized PLS model, listed in Table 1. Since they were expected to be highly relevant to the problem statement, both the C­(O)–H and the C­(N)–H bond dissociation-energy descriptors were selected, despite that the former is missing in Compound **8** from the training set while the latter is missing in Compound **35** in the prediction set. Optimizing the model using the Genetic Algorithm resulted in a reduction of the cross-validation error down to 18.2 J/g, and a considerably better predicted vs measured fit, as evidenced by the data in Figure 6. Applying the optimized PLS model to the prediction set resulted in a reasonably good fit and a standard error of prediction of 95.2 J/g, as shown in Figure 6 (right). As was the case with the unoptimized PLS model, the reactivity of Compound **35** was similarly overestimated, but the overall prediction error was noticeably reduced following the GA model optimization.

**Figure 6:**
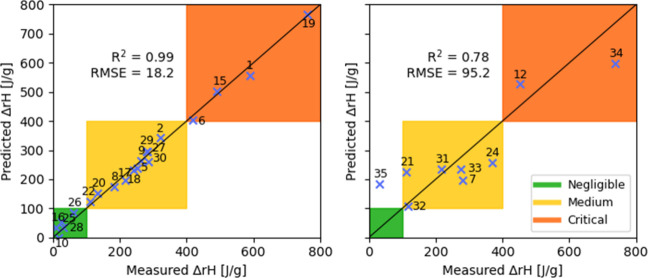
Cross-validation (left) and prediction (right) results obtained using the optimized PLS model, using the optimal set of 16 descriptors.

This result demonstrates the effectiveness of the Genetic Algorithm in selecting the most informative descriptors for modeling the compounds’ reactivity. The very value of the low cross-validation error suggests that the model is overfitted. Indeed, the GA effectively goes through a large multitude of combinations of input features, leading to a model that fits the training set optimally. Nevertheless, despite the apparent overfitting, the model remained robust and delivered accurate predictions, providing reliable preliminary insights into the overall safety risk posed by the sulfamidates.

### 3.3 LASSO Model Results

The LASSO regression selected 17 descriptors out of the full set of 71. Importantly, 12 are in common with those selected by the GA and PLS method, as shown in Table 1. We obtain a SEV of 10.5 J/g and a SEP of 115.7 J/g. The quite pronounced overfitting can be partially explained with the small number of compounds in the training set (20). The LASSO method cannot select more descriptors than the number of observations in the data set, here 20. Therefore, the relative high number of descriptors selected (17 out 20) indicates that the effect of the descriptors is dense rather than sparse, i.e., that all (or a lot of) the descriptors contribute in a non-negligeable way. The value of the hyperparameter λ obtained by the leave-one-out cross validation is quite small, implying that the LASSO regression is getting close to a standard linear regression model.

Despite the apparent overfitting, the LASSO model provided good prediction results and was able to classify 8 out of 9 compounds in the correct safety classes, as shown in Figure 7. The sole misclassified compound was again Compound **35**, with a slightly overestimated reaction enthalpy.

**Figure 7:**
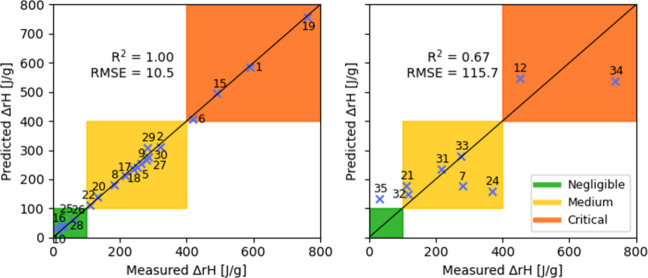
Cross-validation (left) and prediction (right) results obtained using the LASSO method.

### 3.4 Gaussian Process Regression Results

For the Gaussian Process Regression, the features were selected manually based on the Pearson correlation with the reaction enthalpy on the training set. It is reassuring that the features we obtain this way focus on the oxygen atom and the adjacent carbon atom in the ring, relating to the presumed reaction pathway discussed in the Supporting Information. The final set of six selected descriptors is shown in Table 1.

The predictions of the model are shown in Figure 8. Comparing to the other models, the GPR approach shows no overfitting within the cross validation, achieving a SEV of 108.3 J/g and SEP of 101.8 J/g, respectively. This is not due to the model having less variance but rather stems from limited feature selection. The manual process only explored a few combinations of input features, leading to this outcome. Note that the GPR model natively predicts uncertainty intervals, these are shown for the evaluation on the test set. It is interesting to observe that the model is less certain about the predictions of the compounds which have a higher measured reaction enthalpy. These are rarer in the training set, and we see here that the respective regions in the feature space are also covered less by the training set, leading to the higher predicted uncertainties.

**Figure 8:**
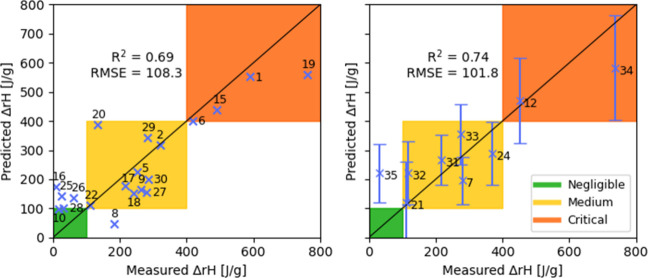
Cross-validation (left) and prediction (right) results obtained using the GPR method.

As for the other models, the model underpredicts the reaction enthalpy for Compound **34**, while it overestimates that of Compound **35**. This leads to all but one compound being classified correctly: Compound **35** is classified as belonging to the medium thermal criticality class, while the measurement shows that its true criticality is negligible. We note that for Compound **35**, the whole uncertainty interval falls into the medium thermal criticality class and that for three other compounds (**12**, **21** and **33**), the uncertainty interval covers more than one criticality class.

### 3.5 Similarities and Differences between the Models

The modeling methods selected a subset of the full set of 71 descriptors, as shown below in Table 1. The full set of descriptors is documented in the Supporting Information.

Overall, the PLS-GA and LASSO methods selected a largely overlapping set of descriptors, with 12 descriptors in common out of the 16 selected by PLS-GA and 17 selected by LASSO, whereas the GPR model utilized a distinctly different set, comprising only 6 descriptors to avoid overfitting.

The difference in the total number of descriptors selected by each method also accounts for the notable variation in cross-validation error values among the three models, with PLS-GA and LASSO showing very low SEV and GPR exhibiting a higher SEV. The overfitting observed with the PLS-GA and LASSO models can be explained by the fact that the regression is done on merely 20 training data points with as many as 16 (respectively 17) explanatory variables (descriptors). The Gaussian Process Regression is done with only 6 explanatory variables, which limits the tendency to overfit.

It is noteworthy that, despite the limited precision of the base model derived solely from DSC measurements, the “DSC Sum dH” descriptor, which was defined using these measurements, was consistently selected by both the PLS-GA and LASSO models. For the GPR model it was not considered due to the low correlation with the reaction enthalpy. This finding underscores the utility of DSC measurements, which are routinely collected in safety laboratories, within our modeling approach. But at the same time, it also demonstrates that relying solely on the DSC measurements to predict thermal reactivity is not enough.

Figure 9 illustrates a comparison of how the nine compounds of the prediction set were classified within the corresponding thermal criticality classes by the three models. It can be observed that the results are similar, with all but one of the compounds correctly classified. As already noted, the criticality of Compound **35** was overestimated by all three models. One potential reason for this could be attributed to the structural dissimilarity of Compound **35** compared to the other compounds characterized by a low reaction enthalpy (Compounds **10**, **16**, **25**, **26**, and **28**). It would seem, therefore, that Compound **35** showed low reaction enthalpy for structural reasons that the models were not trained to identify. Furthermore, it is noteworthy that all three models demonstrate a tendency to limit the range of predictions, steering clear of extreme values (0 and 800 J/g) in their estimated outputs. This aligns with a common tendency of statistical models: when the modeled features offer minimal information about the outcome, the optimal prediction gravitates toward the mean.

**Figure 9:**
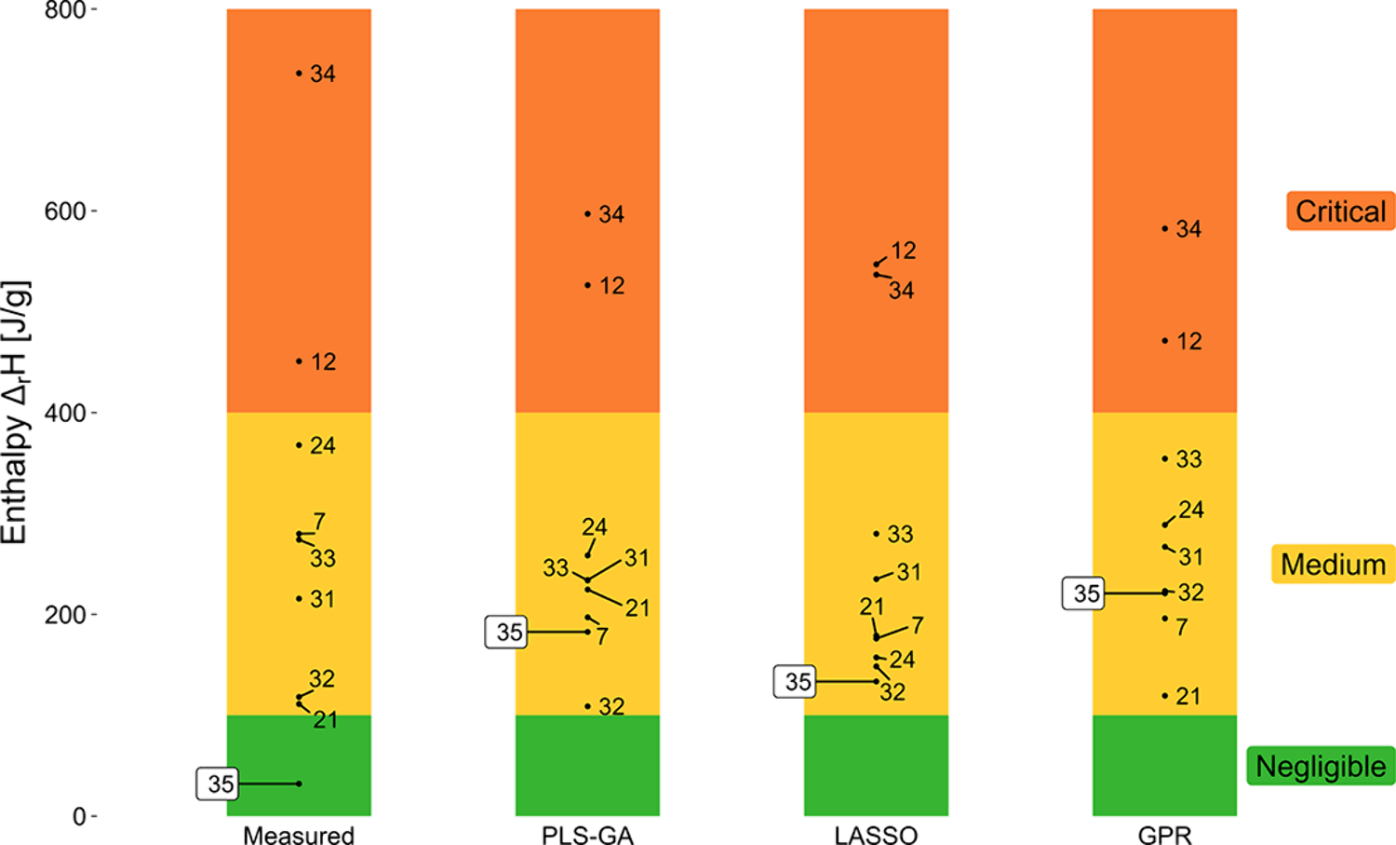
Nine compounds in the prediction set classified according to the thermal criticality class, as defined by Stoessel.^37^ The figure shows a comparison between the measured criticality classes (left) and those predicted by the three models. Compound **35**, whose enthalpy was overestimated by the three models, is highlighted.

The construction of the three models entail varying levels of computational effort. The LASSO and the GPR models are very quick to build, as is the base (unoptimized) PLS model. However, the Genetic Algorithm required to optimize the PLS model takes several hours to converge. Another difference concerns the way that the descriptors are selected. The LASSO and the PLS-GA methods involve an automated, algorithm-based selection of descriptors, whereas the GPR method requires manual selection which, in turn, requires a certain degree of chemical knowledge about the compounds and their reactivity.

### 3.6 Combining Different Models

It is often observed that ensembles of models can achieve more accurate predictions than the single models themselves.^55^ In fact, if the errors of the models are assumed to be independent this statement can be made precise.^56^ In the present case (and as is frequently the case for combinations of models), the errors of the three models are not uncorrelated, note e.g., that all models overpredict Compound **35** while they underpredict compound **34**. In our case, constructing an ensemble of our models does not lead to a significant improvement of the accuracy of the predictions on the prediction set. The results of combining the three models by simply considering the mean of their predictions with equal weights are shown in Table 2, where all possible combinations of models are considered.

**Table 2:** Overview of Model Combinations and Their Performance on the Test Set

PLS-GA	GPR	LASSO	SEP	*R* ^2^
X	X	X	97.4	0.77
X	X		91.9	0.79
X		X	102.9	0.74
	X	X	102.5	0.74
X			95.2	0.78
	X		101.8	0.74
		X	115.7	0.67

The best performance is achieved by combining the PLS-GA and the GPR model, which leads to a SEP of 91.9 J/g, the results are shown in Figure 10.

**Figure 10:**
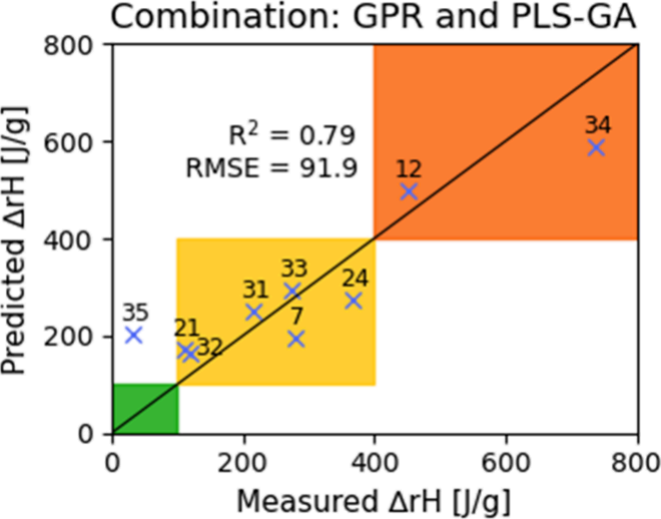
Prediction results obtained using the PLS-GA and GPR modeling methods combined.

Finally, the PLS and LASSO algorithms were evaluated using the set of six descriptors selected for the GPR model, yielding cross-validation and prediction errors of SEV = 118 J/g, SEP = 107 J/g for PLS, and SEV = 96.1 J/g, SEP = 111 J/g for LASSO. The results are provided in the SI data file.

## 4 Conclusions and Perspectives

In this study, we have developed and assessed a statistical modeling methodology for predicting the reactive enthalpy for a specific family of 5-membered cyclic sulfamidates. When applying this methodology to new compounds, it is advisable to conduct a DSC analysis, calculate the relevant descriptors, and then use the model to predict Δ_r_ *H*. Depending on the thermal safety classification – green, yellow, orange or red – appropriate precautions can be taken. This approach not only saves time and reduces costs compared to a full safety analysis but also enhances overall efficiency. While it does not replace comprehensive thermal safety assessments, it serves as a valuable decision-making tool, guiding users toward the necessary analyses and helping streamline the process.

While the error of prediction remains relatively high for the three models (around 100 J/g), all but one of the compounds were classified accurately in the correct risk group. As such, the methodology provides a reliable early safety evaluation approach for process development and scale-up. The objective of this study was to develop and study models supporting preliminary safety assessment and aiding in decision-making. Our focus was not on predicting precise Δ_r_ *H* values, but rather on approximating the release of energy for risk classification purposes. We evaluated three distinct modeling approaches utilizing quantum chemistry-derived descriptors and found that all three methods outperformed the benchmark model, which relied solely on readily available DSC analysis data.

The present study uses at least an order of magnitude less data than other small-data models yet was able to achieve practically useful prediction accuracy by augmenting models with quantum-chemistry derived insights. Only 29 compounds were available for training and testing the model. We have split the compounds to form a training set, composed of 20 samples, and a test set comprising the remaining 9 samples. According to a recent article by Jenkins and Quintana-Ascencio,^57^ a minimum sample size of 25 is required for the training set in order to achieve statistical validity in regression applications involving data with a high degree of variance. Walker et al.^58^ go further to recommend that the ratio of the number of samples to the number of descriptors should be at least 5:1 to minimize the risk of chance correlations. Given the severely limited number of available compounds, maintaining this ratio was not achievable in this study; consequently, we focused on optimally minimizing feature count while preserving sufficient molecular representativeness to ensure model robustness. Additionally, in this study, missing values in both the training and tests sets (the BDE C­(O)–H descriptor in Compound **8** and the BDE C­(N)–H descriptor in Compound **35**) compounded the difficulty in building a robust model. As discussed earlier, we found that substituting these missing data points by the average values of the two missing descriptors provided the best solution. Nevertheless, missing data points are always problematic in statistical learning applications.

Compared to our initial study,^1^ here we have introduced Boltzmann-averaging of thoroughly generated and selected conformer sets for all compounds, explored a larger descriptor space and more diversity of models. The results were robust in all cases and provided consistent and useful prediction accuracies. As in our initial study, our focus remained on a simple and fast thermal hazard and fast safety risk assessment upon change of a protective group in the sulfamidate; keeping in mind that an exact reaction understanding is not given and hence a direct DFT assessment is not practical.

We encourage the scientific community to further explore the superlow data regime: When building models tailored to a specific compound and reaction class, what is the lower limit of suitably selected descriptors and of representative compounds necessary to achieve a robust model with useful prediction accuracy? Further studies of this kind could complement classical ML strategies focusing on maximizing the number of data points, while also sharpening the view on quantum feature engineering and further development of quantum-chemistry based descriptors.

## Supplementary Material





## Data Availability

The data supporting the findings of this study are available within the article and its Supporting Information published online. Source codes are available upon request.

## Notes

Additional information about the methods; centralized data file containing information about the compounds, the descriptors and the results obtained with the methods investigated; and ChemDraw files containing the structures of the compounds (PDF)
Archive containing all the compounds which were considered for the calculations of the descriptors, as well as DBU and SO_3_ and several conformations, in separate sdf files, which contain all calculated properties in the SD tags (ZIP)

## Abbreviations

DSC: Differential Scanning Calorimeter
GA: Genetic Algorithms
GPR: Gaussian Process Regression
LASSO: Least Absolute Shrinkage and Selection Operator
ML: machine learning
PLS: Partial Least Squares
RMSE: root-mean squared error
SEV: standard error of (cross) validation
SEP: standard error of prediction
QC: quantum chemistry

## References

[1] Ferrari T., Blum C., Amini-Rentsch L., Brodard P., Dabros M., Hoehn P., Udvarhelyi A., Marti R., Parmentier M. (2022). Thermal Safety
and Structure-Related Reactivity Investigation of Five-Membered Cyclic
Sulfamidates. Org. Process Res. Dev..

[2] Navo C. D., Mazo N., Avenoza A., Busto J. H., Peregrina J. M., Jiménez-Osés G. (2017). Substituent Effects on the Reactivity
of Cyclic Tertiary Sulfamidates. J. Org. Chem..

[3] Eskici M., Karanfil A., Özer M. S., Sarıkürkcü C. (2011). Reactivity
of cyclic sulfamidates towards lithium acetylides: synthesis of alkynylated
amines. Tetrahedron Lett..

[4] Hammett L. P. (1937). The Effect
of Structure upon the Reactions of Organic Compounds. Benzene Derivatives. J. Am. Chem. Soc..

[5] Williams W. L., Zeng L., Gensch T., Sigman M. S., Doyle A. G., Anslyn E. V. (2021). The Evolution of
Data-Driven Modeling in Organic Chemistry. ACS
Central Science.

[6] Bran A. M., Cox S., Schilter O., Baldassari C., White A. D., Schwaller P. (2024). Augmenting
large language models with chemistry tools. Nat. Mach. Intell..

[7] Götz J., Richards E., Stepek I., Takahashi Y., Huang Y. L., Bertschi L., Rubi B., Bode J. (2024). Predicting
Three-Component Reaction Outcomes from 40k Miniaturized Reactant Combinations. ChemRxiv.

[8] Sanchez-Lengeling B., Aspuru-Guzik A. (2018). Inverse molecular design using machine
learning: Generative
models for matter engineering. Science.

[9] Christensen M., Yunker L. P. E., Adedeji F., Häse F., Roch L. M., Gensch T., dos Passos Gomes G., Zepel T., Sigman M. S., Aspuru-Guzik A., Hein J. E. (2021). Data-science driven autonomous process optimization. Commun. Chem..

[10] Haas B. C., Goetz A. E., Bahamonde A., McWilliams J. C., Sigman M. S. (2022). Predicting relative efficiency of
amide bond formation
using multivariate linear regression. Proc.
Natl. Acad. Sci. U. S. A..

[11] Guan Y., Coley C. W., Wu H., Ranasinghe D., Heid E., Struble T. J., Pattanaik L., Green W. H., Jensen K. F. (2021). Regio-selectivity prediction with
a machine-learned reaction representation and on-the-fly quantum mechanical
descriptors. Chemical Science.

[12] Shimakawa H., Kumada A., Sato M. (2024). Extrapolative prediction of small-data
molecular property using quantum mechanics-assisted machine learning. npj Comput. Mater..

[13] Finkelmann A. R., Göller A. H., Schneider G. (2017). Site of Metabolism Prediction Based
on ab initio Derived Atom Representations. ChemMedChem..

[14] Ertl P., Gerebtzoff G., Lewis R., Muenkler H., Schneider N., Sirockin F., Stiefl N., Tosco P. (2022). Chemical Reactivity
Prediction: Current Methods and Different Application Areas. Mol. Inf..

[15] Gandhi S. S., Brown G. Z., Aikonen S., Compton J. S., Neves P., Martinez Alvarado J. I., Strambeanu I. I., Leonard K. A., Doyle A. G. (2025). Data Science-Driven
Discovery of Optimal Conditions and a Condition-Selection Model for
the Chan-Lam Coupling of Primary Sulfonamides. ACS Catal..

[16] Šícho M., de Bruyn Kops C., Stork C., Svozil D., Kirchmair J. (2017). FAME 2: Simple
and Effective Machine Learning Model of Cytochrome P450 Regioselectivity. J. Chem. Inf. Model..

[17] Keto A., Guo T., Underdue M., Stuyver T., Coley C. W., Zhang X., Krenske E. H., Wiest O. (2024). Data-Efficient, Chemistry-Aware Machine
Learning Predictions of Diels-Alder Reaction Outcomes. J. Am. Chem. Soc..

[18] Strieth-Kalthoff F., Sandfort F., Segler M. H. S., Glorius F. (2020). Machine learning the
ropes: principles, applications and directions in synthetic chemistry. Chem. Soc. Rev..

[19] Duan W., Pan Y., He H., Zhao S., Zhao X., Jiang J., Shu C.-M. (2020). Prediction
of the thermal decomposition temperatures
of imidazolium ILs based on norm indexes. J.
Mol. Liq..

[20] Sayyed F. B., Kolis S. P., Xia H. (2022). Quantum Mechanical Methods for Thermal
Hazard Risk Assessment in Early Phase Pharmaceutical Development. Org. Process Res. Dev..

[21] Blum C., Amini-Rentsch L., Ferrari F. T., Brodard P., Marti R., Hoehn P., Dabros M., Parmentier M. (2022). Development,
Validation, and Application of a Custom-Made Mini- Reaction Calorimeter
for Thermal Safety Screening. Org. Process Res.
Dev..

[22] Jiménez-Osés G., Avenoza A., Busto J. H., Peregrina J. M. (2008). Highly
chemoselective reactions on hindered sulfamidates with oxygenated
nucleophiles. Tetrahedron: Asymmetry.

[23] Bower J. F., Rujirawanich J., Gallagher T. (2010). N-Heterocycle construction via cyclic
sulfamidates. Applications in synthesis. Organic
& Biomolecular Chemistry.

[24] Hill S. A., Steinfort R., Mücke S., Reifenberger J., Sengpiel T., Hartmann L. (2021). Exploring
Cyclic Sulfamidate Building
Blocks for the Synthesis of Sequence-Defined Macromolecules. Macromol. Rapid Commun..

[25] Avenoza A., Busto J. H., Corzana F., Jiménez-Osés G., Peregrina J. M. (2004). SN2 vs. E2 on quaternary centres: an application to
the synthesis of enantiopure β2,2-amino acids. Chem. Commun..

[26] Mata L., Avenoza A., Busto J. H., Peregrina J. M. (2013). Chemoselectivity
Control in the Reactions of 1,2-Cyclic Sulfamidates with Amines. Chemistry
– A. European Journal.

[27] Brethomé A. V., Fletcher S. P., Paton R. S. (2019). Conformational
Effects on Physical-Organic
Descriptors: The Case of Sterimol Steric Parameters. ACS Catal..

[28] Finkelmann A. R., Göller A. H., Schneider G. (2016). Robust molecular representations
for modelling and design derived from atomic partial charges. Chem. Commun..

[29] Udvarhelyi A., Rodde S., Wilcken R. (2021). ReSCoSS: a
flexible quantum chemistry
workflow identifying relevant solution conformers of drug-like molecules. J. Comput. Aided Mol. Des..

[30] Bannwarth C., Caldeweyher E., Ehlert S., Hansen A., Pracht P., Seibert J., Spicher S., Grimme S. (2021). Extended tight-binding
quantum chemistry methods. Wiley Interdiscip.
Rev.: Comput. Mol. Sci..

[31] Furche F., Ahlrichs R., Hättig C., Klopper W., Sierka M., Weigend F. (2014). Turbomole. WIREs Computational
Molecular Science.

[32] Bochevarov A. D., Harder E., Hughes T. F., Greenwood J. R., Braden D. A., Philipp D. M., Rinaldo D., Halls M. D., Zhang J., Friesner R. A. (2013). Jaguar: A high-performance quantum
chemistry software program with strengths in life and materials sciences. Int. J. Quantum Chem..

[33] Parr R. G., Yang W. (1984). Density functional
approach to the frontier-electron theory of chemical
reactivity. J. Am. Chem. Soc..

[34] Domingo L. R., Ríos-Gutiérrez M., Pérez P. (2016). Applications
of the Conceptual Density Functional Theory Indices to Organic Chemistry
Reactivity. Molecules.

[35] Danilack A. D., Dickson C. J., Soylu C., Fortunato M., Rodde S., Munkler H., Hornak V., Duca J. S. (2024). Reactivities
of acrylamide warheads toward cysteine targets: a QM/ML approach to
covalent inhibitor design. J. Comput. Aided
Mol. Des..

[36] St.
John P. C., Guan Y., Kim Y., Kim S., Paton R. S. (2020). Prediction of organic homolytic bond dissociation enthalpies
at near chemical accuracy with sub-second computational cost. Nat. Commun..

[37] Stoessel, F. Thermal Safety of Chemical Processes: Risk Assessment and Process Design; Wiley-VCH Verlag GmbH & Co.: 2020.

[38] Wold S., Sjöström M., Eriksson L. (2001). PLS-regression: a basic
tool of chemometrics. Chemometrics Intellig.
Lab. Syst..

[39] Brereton, R. G. Calibration. In Applied Chemometrics for Scientists; John Wiley & Sons, L.td: 2007; pp 193–220.

[40] Martens, H. ; Næs, T. Multivariate Calibration; John Wiley & Sons, Ltd: 1989.

[41] Katritzky A. R., Kuanar M., Slavov S., Hall C. D., Karelson M., Kahn I., Dobchev D. A. (2010). Quantitative
Correlation of Physical
and Chemical Properties with Chemical Structure: Utility for Prediction. Chem. Rev..

[42] Hassanat A., Almohammadi K., Alkafaween E., Abunawas E., Hammouri A., Prasath V. B. S. (2019). Choosing
Mutation and Crossover Ratios for Genetic
Algorithms-A Review with a New Dynamic Approach. Information.

[43] Bingul, Z. ; Sekmen, A. S. ; Palaniappan, S. ; Zein-Sabatto, S. Genetic algorithms applied to real time multiobjective optimization problems. In Proceedings of the IEEE SoutheastCon 2000. ‘Preparing for The New Millennium’ (Catal. No.00CH37105); IEEE: 2000; pp 95–103.

[44] Gutowski, M. Biology, Physics, Small Worlds and Genetic Algorithms. In Leading Edge Computer Science Research; Nova Science Publishers: 2005; pp 165–218.

[45] Bangalore A. S., Shaffer R. E., Small G. W., Arnold M. A. (1996). Genetic Algorithm-Based
Method for Selecting Wavelengths and Model Size for Use with Partial
Least-Squares Regression: Application to Near-Infrared Spectroscopy. Anal. Chem..

[46] Duraipandian S., Zheng W., Ng J., Low J. J. H., Ilancheran A., Huang Z. (2011). In vivo diagnosis of
cervical precancer using Raman spectroscopy
and genetic algorithm techniques. Analyst.

[47] Leardi R., Seasholtz M. B., Pell R. J. (2002). Variable selection for multivariate
calibration using a genetic algorithm: prediction of additive concentrations
in polymer films from Fourier transform-infrared spectral data. Anal. Chim. Acta.

[48] Salim M. M., El Sharkasy M. E., Walash M., Belal F. (2020). Genetic Algorithm with
Model-Updating-Based PLS Regression for the Spectrophotometric Determination
of Clopidogrel, Atorvastatin, and Aspirin in the Presence of its Degradation
Product. J. Appl. Spectrosc..

[49] Butkus M., Repšytė J., Galvanauskas V. (2020). Fuzzy Logic-Based
Adaptive Control of Specific Growth Rate in Fed-Batch Biotechnological
Processes. A Simulation Study. Appl. Sci..

[50] Tibshirani R. (1996). Regression
shrinkage and selection via the Lasso. Journal
of the Royal Statistical Society Series B-Statistical Methodology.

[51] Yerly F., Blaise M., Barras S. (2023). Machine Learning Models for Melting
Point Prediction of Ionic Liquids: CatBoost Approach: FH-HES Universities
of Applied Sciences. CHIMIA.

[52] Friedman J. H., Hastie T., Tibshirani R. (2010). Regularization
Paths for Generalized
Linear Models via Coordinate Descent. J. Stat.
Software.

[53] Beckers T. (2021). An Introduction
to Gaussian Process Models. ArXiv.

[54] Pedregosa F., Varoquaux G., Gramfort A., Michel V., Thirion B., Grisel O., Blondel M., Prettenhofer P., Weiss R., Dubourg V., Vanderplas J., Passos A., Cournapeau D., Brucher M., Perrot M., Duchesnay É. (2011). Scikit-learn: Machine Learning in Python. J. Mach. Learn. Res..

[55] Carlens, H. State of Competitive Machine Learning in 2022. In ML Contests Research [Online], 2023. https://mlcontests.com/state-of-competitive-machine-learning-2022.

[56] Bishop, C. M. 14. Combining Models. In Pattern recognition and machine learning; Springer: New York, 2006.

[57] Jenkins D. G., Quintana-Ascencio P. F. (2020). A solution to minimum sample size for regressions. PLoS One.

[58] Walker J. D., Jaworska J., Comber M. H. I., Schultz T. W., Dearden J. C. (2003). Guidelines
for developing and using quantitative structure-activity relationships. Environ. Toxicol. Chem..

