# Supplementary material for: Quantum Descriptor-Based Machine-Learning Modeling of Thermal Hazard of Cyclic Sulfamidates
Source: J Chem Inf Model. 2025 Aug 15;65(16):8624–36. doi: 10.1021/acs.jcim.5c01048 (PMC12381854; doi:10.1021/acs.jcim.5c01048)
Supplement: Supplementary file 2 [file ci5c01048_si_002.zip › Novartis_MVA - REVISED SI.docx]

**Quantum descriptor-based machine-learning modelling of thermal hazard of cyclic sulfamidates**

Michal Dabros^*^, Hagen Münkler, Florence Yerly, Roger Marti, Michaël Parmentier and Anikó Udvarhelyi^*^

**Supplementary Information**

**1. INTRODUCTION**

In the Supplementary Information, we provide additional details about the calculation of the quantum mechanical descriptors that were used to build the models. In addition to the explanations below, we provide SDF files which contain the structures of the compounds and degradants as well as all calculated quantum mechanical descriptors for them. The descriptors are summarized in an excel file giving all the descriptors of each compound, which arise after Boltzmann-averaging the properties noted in the SDF files. Moreover, the Pearson correlation coefficients to the reaction enthalpy and an overview table of the calculation methods are provided.

**2. COMPLETE LIST OF COMPOUNDS**

As mentioned in the main text, a major limitation for the modelling approach was the small dataset size of only 29 compounds. This was due to synthesis challenges and scarce commercial availability of eligible compounds. Figure S1 presents the complete list of compounds that were considered, along with the synthesis conditions and the yields. Compounds **3**, **4**, **11**, **13**, **14** and **23** could not be synthesized.

Figure S1. Complete list of sulfamidates considered in the study, along with synthesis routes and yield values. Compounds **3**, **4**, **11**, **13**, **14** and **23** could not be synthesized.

**3. COMPOUND DESCRIPTORS**

We aim to provide a diverse set of descriptors which can capture the reactivity of the compound with respect to the reaction shown in Figure 2 in the main text. Due to the common five-membered ring, we have a straightforward way of incorporating atom- or bond-specific descriptors which are related to the atoms within this ring. Overall, we can thus describe each compound as a feature vector which comprises some descriptors of the whole molecule, as well as atom- and bond-specific descriptors located on specific atoms or bonds in the common ring of the cyclic sulfamidates.

As part of the supplementary information, we provide an excel file, which contains an overview of the calculation methods, the values of all calculated descriptors for each compound and the Pearson correlation with the reaction enthalpies. The overview of the descriptor calculations is also given in Table S1 below.

For all calculations, we rely on the ReSCoSS workflow^1^ to generate a set of conformers for all compounds. The final geometry optimization in the ReSCoSS workflow is done at the level b3lyp/def2-TZVP. The only descriptor with a different level of geometry optimization are the bond dissociation energies for the hydrogens connected to the atoms C(O) and C(N) in the five-membered ring, for which the jaguar workflow^2^ uses a geometry optimization at the level b3lyp/LACVP*.

The molecular properties are then calculated for each conformer in the set, and the descriptors are obtained by calculating the Boltzmann-weighted average over the conformers. The Boltzmann weights are determined from the energies of the different conformers in acetonitrile, which are calculated using Cosmotherm.^3^

All these calculations, from the conformer expansion to each of the QM property calculations, are orchestrated using an internal Novartis python package.

Table S1. Overview of QM-calculated descriptors which were considered for the modelling of reaction enthalpies

| **Descriptor** | **Type** | **Software** | **Level of theory: Geometry** | **Level of theory: Property Calculation** | **solution model** | **#** |
| --- | --- | --- | --- | --- | --- | --- |
| Fukui index f plus | atom-specific | xtb | b3lyp/def2-TZVP | semi-empirical (GFN2-xtb) | Acetonitrile(alpb) | 8 |
| Fukui index f minus | atom-specific | xtb | b3lyp/def2-TZVP | semi-empirical (GFN2-xtb) | Acetonitrile(alpb) | 8 |
| Fukui index f zero | atom-specific | xtb | b3lyp/def2-TZVP | semi-empirical (GFN2-xtb) | Acetonitrile(alpb) | 8 |
| Mulliken charges | atom-specific | xtb | b3lyp/def2-TZVP | semi-empirical (GFN2-xtb) | Acetonitrile(alpb) | 8 |
| Fukui Index (NN) HOMO | atom-specific | Jaguar | b3lyp/def2-TZVP | b3lyp-d3/LACVP* | gas phase | 8 |
| Fukui Index (NN) LUMO | atom-specific | Jaguar | b3lyp/def2-TZVP | b3lyp-d3/LACVP* | gas phase | 8 |
| Labute's Approximate Surface Area | atom-specific | rdkit | - | - | - | 3 |
| Visible sky | atom-specific | own code | b3lyp/def2-TZVP | - | - | 5 |
| Bond Dissociation Energy | bond-specific | Jaguar | b3lyp/LACVP* | M06-2X/cc-pVTZ(-f) | gas phase | 2 |
| Bond order | bond-specific | xtb | b3lyp/def2-TZVP | semi-empirical (GFN2-xtb) | Acetonitrile(alpb) | 1 |
| Global electro-philicity index | molecule-wide | xtb | b3lyp/def2-TZVP | semi-empirical (GFN2-xtb) | Acetonitrile(alpb) | 3 |
| Dipole moment | molecule-wide | xtb | b3lyp/def2-TZVP | semi-empirical (GFN2-xtb) | Acetonitrile(alpb) | 1 |
| Homo-Lumo gap | molecule-wide | xtb | b3lyp/def2-TZVP | semi-empirical (GFN2-xtb) | Acetonitrile(alpb) | 3 |
| Sum of Peaks DSC | molecule-wide | experiment | - | - | - | 1 |
| Molecular weight | molecule-wide | rdkit | - | - | - | 2 |
| Fukui index f plus | atom-specific | Turbomole | b3lyp/def2-TZVP | b3lyp/def2-TZVPD | Acetonitrile(cosmo) | 8 |
| Fukui index f minus | atom-specific | Turbomole | b3lyp/def2-TZVP | b3lyp/def2-TZVPD | Acetonitrile(cosmo) | 8 |
| Fukui index f zero | atom-specific | Turbomole | b3lyp/def2-TZVP | b3lyp/def2-TZVPD | Acetonitrile(cosmo) | 8 |
| nmr shielding constants | atom-specific | Turbomole | b3lyp/def2-TZVP | b3lyp/def2-TZVPD | Acetonitrile(cosmo) | 8 |
| Mulliken charges | atom-specific | Turbomole | b3lyp/def2-TZVP | b3lyp/def2-TZVPD | Acetonitrile(cosmo) | 8 |
| Electron affinity | molecule-wide | Turbomole | b3lyp/def2-TZVP | b3lyp/def2-TZVPD | Acetonitrile(cosmo) | 1 |
| Electronegativity | molecule-wide | Turbomole | b3lyp/def2-TZVP | b3lyp/def2-TZVPD | Acetonitrile(cosmo) | 1 |
| Electrophilicity | molecule-wide | Turbomole | b3lyp/def2-TZVP | b3lyp/def2-TZVPD | Acetonitrile(cosmo) | 1 |
| Hardness | molecule-wide | Turbomole | b3lyp/def2-TZVP | b3lyp/def2-TZVPD | Acetonitrile(cosmo) | 1 |
| Ionization potential | molecule-wide | Turbomole | b3lyp/def2-TZVP | b3lyp/def2-TZVPD | Acetonitrile(cosmo) | 1 |
| Free energy difference | molecule-wide | Turbomole | b3lyp/def2-TZVP | b3lyp/def2-TZVPD | gas phase | 2 |

Given these geometries, we calculate the following atom-specific properties of the molecules:

- First, we calculate partial charges for the different atoms, using both GFN2-xtb^4^ and Turbomole.^5^ In either case, we are relying on the Mulliken scheme to condense the electron distribution into partial charges assigned to each atom in the compound. The DFT calculation is carried out at the level b3lyp/def2-TZVPD.
- In addition, we calculate Fukui indices. The Fukui index^6-7^ describes how the electron density of a molecule reacts to ionization and is commonly considered when modelling reactivity-related problems^8-9^. Specifically, the electron densities of the molecule with varying charges are compared to compute the electrophilic (0 and –1), nucleophilic (0 and +1) and radical (+1 and –1) Fukui indices. While GFN2-xtb and Turbomole rely on a similar approach to define the Fukui indices, Jaguar uses a different approach, which allows to extract the Fukui indices from the neutral state directly^10-11^. In this approach, jaguar differentiates partial derivatives of the electron and spin density using the subscripts N and S, respectively. We focus on the “NN” version of these, which only refer to the electron density.
- Additionally, we consider NMR shielding constants. The electron density has an influence on the shielding of the nuclei with respect to electromagnetic radiation, which is reflected in the NMR shielding constants. They thus represent another way to encode information about the electronic density. We rely on Turbomole for the calculation of the NMR shielding constants.

All these descriptors are collected for each heavy atom in the ring displayed in Figure 2a in the main text as well as for the hydrogen atoms which are bonded to them. In case there are two hydrogens bonded to the respective atom, the property is averaged over them, if there are no hydrogens, we use the mean value of the respective descriptor for the compounds which are in the training set. The motivation for this handling of missing values is given in section 2.3 in the main text.

Additionally, we have considered atom-specific descriptors which are of purely geometrical nature and give information on the accessibility of the atom in question, i.e. the steric hindrance of the reaction. Concretely, we consider the following descriptors:

- Labute's accessible surface area (ASA), as implemented in RDKit.^12^ This descriptor estimates, based only on the graph structure, the solvent accessible surface area of a molecule, we are considering each individual atom's contribution.
- Visible sky descriptor: We can also describe the accessibility of a single atom by considering the three-dimensional conformations of the molecules which we have generated. Here, we relied on our own implementation, which calculates which part of the full solid angle is obstructed by other atoms when looking from the centre of the atom in question. The implementation constructs a large set of rays going outward from the centre of the query atom and counts which of these intersect another atom in the molecule. We ensure that the set of rays is constructed in a rotation-invariant fashion. To check whether such a ray intersects an atom, we rely on the van-der-Waals radii of these atoms as provided by the RDKit.

Moreover, we consider two QM-calculated properties which are related to bonds within the common ring of the cyclic sulfamidates:

- Bond dissociation energy: We consider the energy required to dissociate the bond between the two carbon atoms in the common ring and any of its hydrogen atoms. The jaguar software^2^ provides an automatic workflow to calculate this bond dissociation energy.
- For the bonds within the ring depicted in Figure 2a in the main text, we additionally consider the bond order, which is calculated using GFN2-xtb and gives an indication of the strength of the bond.

Additionally, we consider quantum chemical properties of the whole molecule as descriptors. Here, we calculate the dipole moment, global electrophilicity index and homo-lumo gap using GFN2-xtb and five additional properties using the Turbomole Fukui script: The electron affinity ($ea$), the electronegativity ($\chi$), electrophilicity ($\omega$), the hardness ($\eta$) and the ionization potential ($ip$). These are not independent; we note the relations:

$${\eta= \left( ip - ea \right)}/2$$

$${\chi= \left( ip + ea \right)}/2$$

$${\omega= \chi^{2}}/{2\eta}$$

Last, we consider descriptors related to the two degradants shown in the nucleophilic reaction pathway in Figure 2b in the main text. In addition to the homo-lumo gap and the global electrophilicity index of the two degradants, we compare the free energy of the original compound with that of the degradants. To calculate the electronic energy $E$and the chemical potential $\mu$, we rely on the Turbomole freeh script. For each compound and degradant, we thus obtain the free energy $G = E + \mu$. Denoting the two degradants as $d_{1}$ and $d_{2}$ respectively, we thus have the free energy differences:

$$dG_{1}=G\left( d_{1} \right)-G\left( DBU \right)-G\left( cpd \right)$$

$$dG_{2}=G\left( d_{2} \right)+G\left( SO_{3} \right)-G\left( d_{2} \right)$$

**3.1 Reducing the Number of Descriptors.** Proceeding in the way described above, we obtain a set of 116 descriptors for each compound. Note that most atom-dependent properties contribute eight descriptors. While all our methods rely on some feature-reduction mechanism, we decided to reduce the set of descriptors before starting the modelling work. Note that the set of descriptors contains partial charges and Fukui indices calculated using both turbomole and xtb, which should in principle provide the same information to the model. To choose one of the methods, we considered the Pearson correlation of the respective descriptors with the reaction enthalpy in kcal/mol. The six descriptors with the highest correlation are all calculated using xtb; and we hence did not include the turbomole-based descriptors in the set of descriptors used during the modelling work. This is convenient also from the perspective of the calculation time, which is significantly shorter for xtb.

**REFERENCES**

1. Udvarhelyi, A.; Rodde, S.; Wilcken, R., ReSCoSS: a flexible quantum chemistry workflow identifying relevant solution conformers of drug-like molecules. *J. Comput. Aided Mol. Des.* **2021,** *35* (4), 399-415.

2. Bochevarov, A. D.; Harder, E.; Hughes, T. F.; Greenwood, J. R.; Braden, D. A.; Philipp, D. M.; Rinaldo, D.; Halls, M. D.; Zhang, J.; Friesner, R. A., Jaguar: A high-performance quantum chemistry software program with strengths in life and materials sciences. *Int. J. Quantum Chem* **2013,** *113* (18), 2110-2142.

3. Klamt, A.; Eckert, F., COSMO-RS: a novel and efficient method for the a priori prediction of thermophysical data of liquids. *Fluid Phase Equilib.* **2000,** *172* (1), 43-72.

4. Bannwarth, C.; Caldeweyher, E.; Ehlert, S.; Hansen, A.; Pracht, P.; Seibert, J.; Spicher, S.; Grimme, S., Extended tight-binding quantum chemistry methods. *WIREs Computational Molecular Science* **2021,** *11* (2), e1493.

5. Furche, F.; Ahlrichs, R.; Hättig, C.; Klopper, W.; Sierka, M.; Weigend, F., Turbomole. *WIREs Computational Molecular Science* **2014,** *4* (2), 91-100.

6. Parr, R. G.; Yang, W., Density functional approach to the frontier-electron theory of chemical reactivity. *J. Am. Chem. Soc.* **1984,** *106* (14), 4049-4050.

7. Domingo, L. R.; Ríos-Gutiérrez, M.; Pérez, P. Applications of the Conceptual Density Functional Theory Indices to Organic Chemistry Reactivity *Molecules* [Online], 2016.

8. Danilack, A. D.; Dickson, C. J.; Soylu, C.; Fortunato, M.; Rodde, S.; Munkler, H.; Hornak, V.; Duca, J. S., Reactivities of acrylamide warheads toward cysteine targets: a QM/ML approach to covalent inhibitor design. *J. Comput. Aided Mol. Des.* **2024,** *38* (1), 21.

9. Guan, Y.; Coley, C. W.; Wu, H.; Ranasinghe, D.; Heid, E.; Struble, T. J.; Pattanaik, L.; Green, W. H.; Jensen, K. F., Regio-selectivity prediction with a machine-learned reaction representation and on-the-fly quantum mechanical descriptors. *Chemical Science* **2021,** *12* (6), 2198-2208.

10. Contreras, R. R.; Fuentealba, P.; Galván, M.; Pérez, P., A direct evaluation of regional Fukui functions in molecules. *Chem. Phys. Lett.* **1999,** *304* (5), 405-413.

11. Chamorro, E.; Pérez, P., Condensed-to-atoms electronic Fukui functions within the framework of spin-polarized density-functional theory. *The Journal of Chemical Physics* **2005,** *123* (11), 114107.

12. RDKit: *Open-source cheminformatics.* [*https://www.rdkit.org*](https://www.rdkit.org), Release_2023_09_3; Zenodo: 2023.
