# Supplementary figures and images for: Quantum Descriptor-Based Machine-Learning Modeling of Thermal Hazard of Cyclic Sulfamidates
Source: J Chem Inf Model. 2025 Aug 15;65(16):8624–36. doi: 10.1021/acs.jcim.5c01048 (PMC12381854; doi:10.1021/acs.jcim.5c01048)

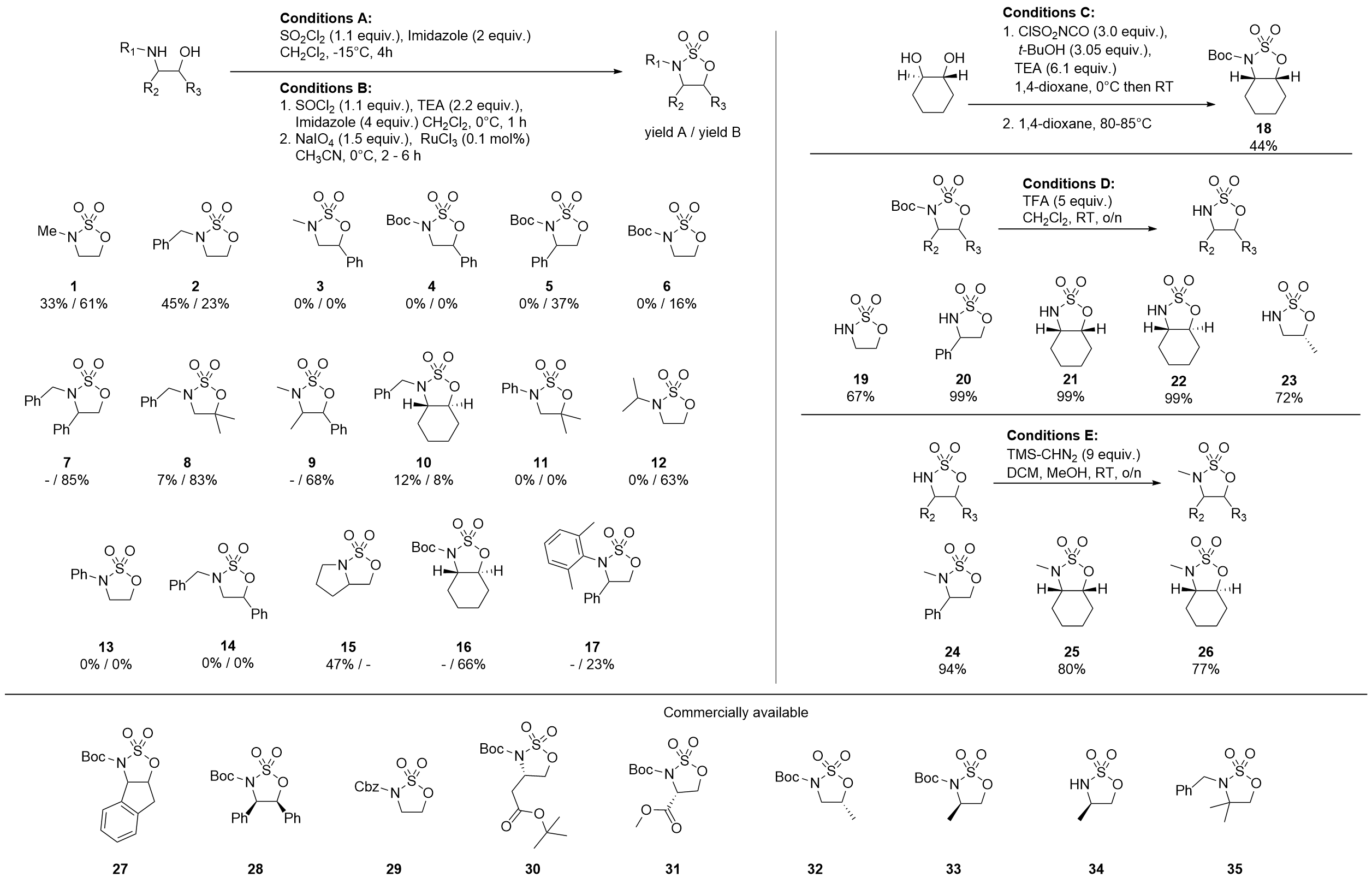

Supplement: Supplementary file 2 [file ci5c01048_si_002.zip › Novartis_MVA - SI - Figure_S1.tif]
